# The Oxidosqualene Cyclase from the Oomycete *Saprolegnia parasitica* Synthesizes Lanosterol as a Single Product

**DOI:** 10.3389/fmicb.2016.01802

**Published:** 2016-11-09

**Authors:** Paul Dahlin, Vaibhav Srivastava, Vincent Bulone, Lauren S. McKee

**Affiliations:** ^1^Division of Glycoscience, School of Biotechnology, KTH Royal Institute of TechnologyStockholm, Sweden; ^2^Department of Ecology, Environment and Plant Sciences, Stockholm UniversityStockholm, Sweden; ^3^ARC Centre of Excellence in Plant Cell Walls, School of Agriculture, Food and Wine, The University of Adelaide, UrrbraeSA, Australia; ^4^Wallenberg Wood Science Centre, KTH Royal Institute of TechnologyStockholm, Sweden

**Keywords:** lanosterol biosynthesis, oomycete, *Saprolegnia parasitica*, oxidosqualene cyclase, sterols

## Abstract

The first committed step of sterol biosynthesis is the cyclisation of 2,3-oxidosqualene to form either lanosterol (LA) or cycloartenol (CA). This is catalyzed by an oxidosqualene cyclase (OSC). LA and CA are subsequently converted into various sterols by a series of enzyme reactions. The specificity of the OSC therefore determines the final composition of the end sterols of an organism. Despite the functional importance of OSCs, the determinants of their specificity are not well understood. In sterol-synthesizing oomycetes, recent bioinformatics, and metabolite analysis suggest that LA is produced. However, this catalytic activity has never been experimentally demonstrated. Here, we show that the OSC of the oomycete *Saprolegnia parasitica*, a severe pathogen of salmonid fish, has an uncommon sequence in a conserved motif important for specificity. We present phylogenetic analysis revealing that this sequence is common to sterol-synthesizing oomycetes, as well as some plants, and hypothesize as to the evolutionary origin of some microbial sequences. We also demonstrate for the first time that a recombinant form of the OSC from *S. parasitica* produces LA exclusively. Our data pave the way for a detailed structural characterization of the protein and the possible development of specific inhibitors of oomycete OSCs for disease control in aquaculture.

## Introduction

**FIGURE 1:**
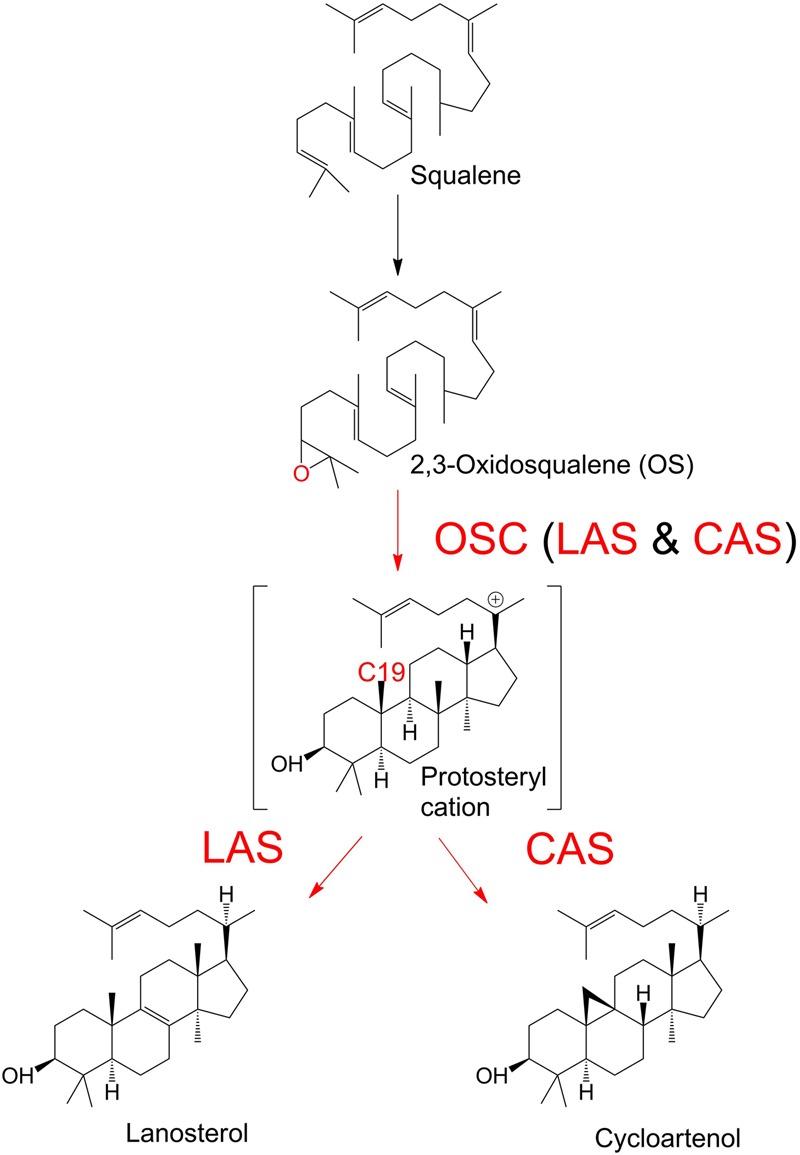
**Enzymatic cyclisation of 2,3-oxidosqualene (OS) to lanosterol (LA) and cycloartenol (CA).** Oxidosqualene cyclase enzymes (OSC) protonate the OS substrate, leading to the production of LA or CA.

The sterols are a diverse group of organic compounds with indispensable structural and biochemical roles in multicellular organisms (Summons et al., 2006; Desmond and Gribaldo, 2009). The first committed step of sterol biosynthesis is the cyclisation of OS to either LA or CA, depending on the organism (Summons et al., 2006; Xue et al., 2012). The product of this decisive biochemical step, catalyzed by an OSC, is subjected to multiple subsequent modifications, which ultimately determine the profile of end sterols. OSC enzymes are classed as CAS or LAS, depending on the outcome of the reactions they catalyze, i.e., the formation of CA or LA (**Figure 1**). The OSC reaction begins with the protonation of the epoxide ring of OS (Corey et al., 1997). This initiates a ring-forming cascade which proceeds through a series of carbocation intermediates before the final stable product forms (Woodward and Bloch, 1953; Thoma et al., 2004). LAS and CAS reactions differ in the later mechanistic stages of the reaction. The formation of a double bond between C8 and C9 will lead to LA production. Conversely, a deprotonation at C19 will cause the cyclopropyl ring to close, and CA will form instead (Abe et al., 1993; Abe, 2007; Ohyama et al., 2009). LA and CA are found in cellular membranes (Briolay et al., 2009). Their biochemistry has been an area of interest for decades, in fields ranging from human medicine to plant physiology (Dean et al., 1967; Huff and Telford, 2005; Ohyama et al., 2009). The primary function of LA and CA in most organisms is to serve as intermediates in sterol synthesis, as they are precursors of the dominant end sterols. Sterols serve a wide range of biochemical and structural roles in diverse organisms, and are involved in hormone production and membrane integrity among many other examples (Summons et al., 2006).

Sterol synthesis pathways involving CA and LA have traditionally been considered to be common to specific kingdoms of life: CA is found in plants, while LA is found in animals and fungi. However, it is now apparent that OSCs cannot be categorized along such simple lines. The mechanisms underlying the specificity of the OSC reaction within and across kingdoms are still not fully established. For example, LAS genes have been identified in several plants (Kolesnikova et al., 2006; Suzuki et al., 2006; Ohyama et al., 2009) and the model dicot *Arabidopsis thaliana* has functional complementary LA and CA biosynthesis pathways, both contributing to the synthesis of a mixture of sterols (Ohyama et al., 2009).

At the level of amino acid sequence, CAS and LAS enzymes are highly similar, and the specific outcome of the cyclisation reaction depends on very small differences in these enzymes. Three specific amino acids differ between CAS and LAS enzymes. In the human LAS, these are at positions 381, 449, and 453. Point mutations at these positions (or their equivalents in homologous proteins) affect the C19 deprotonation step of the reaction (Meyer et al., 2002; Segura et al., 2002; Sawai et al., 2006; Summons et al., 2006; and references therein). This trio of residues found in the C-terminal domain of OSCs invariably consists of Y, H, and I in CAS enzymes. LAS enzymes show more variability, with T or Y in the first position, followed typically by C, Q, or H in the second position, and V in the third position (Summons et al., 2006). The OSC of the oomycete *Saprolegnia parasitica*, an opportunistic fish pathogen responsible for the devastating salmonid disease saprolegniasis, possesses a rare Y,N,V triad that has previously been identified only in some plant LAS enzymes (Kolesnikova et al., 2006; Suzuki et al., 2006).

It was originally suggested that sterol synthesis in the oomycetes proceeded via the formation of CA (Warner and Domnas, 1981; Warner et al., 1982, 1983), but it is increasingly the consensus view that sterol-synthesizing oomycetes in fact produce LA (Nes et al., 1986; Madoui et al., 2009; Jiang et al., 2013; Warrilow et al., 2014). LA has been identified as one of the major sterols in the mycelium of *S. parasitica*, where CA has not been detected (Warrilow et al., 2014). The oomycete has two putative OSC genes (SPRG_11783 and SPRG_17895, identified by BLAST analysis), which are therefore predicted to encode LAS enzymes (Jiang et al., 2013). The two genes are almost identical, except that the N-terminal part of the predicted product of SPRG_17895 is significantly truncated (Warrilow et al., 2014). Both gene products possess the Y,N,V triad that is believed to determine enzyme specificity. It is, however, unclear if both proteins are catalytically active enzymes, as there is limited information on the other protein features required for LAS activity.

As no oomycete OSCs have been biochemically characterized to date, we were motivated to undertake a thorough analysis of the *S. parasitica* OSC to obtain a clear understanding of OS cyclisation in this oomycete, and gain insights into the sterol synthesis pathway. In the longer term this fundamental knowledge may open up new avenues of inhibitor development for disease control in aquaculture by specifically targeting the sterol biosynthetic pathway of the fish pathogen.

## Materials and Methods

All chemicals and reagents were obtained from Sigma Aldrich (St Louis, MO, USA) unless otherwise indicated.

### Bioinformatic Analyses

The predicted LAS genes of *S. parasitica* were aligned against 63 predicted OSC sequences selected from various taxa (Supplementary Table S1). Potential OSCs were selected based on published studies of enzyme characterization, the Brenda database (Chang et al., 2015), and/or Blast analysis using the NCBI database^1^. For phylogenetic analysis in diverse organisms, we only used the catalytic domains of the sequences. The phylogeny software PhyML (version 3.1, Guindon et al., 2010) was used to generate a phylogenetic tree by means of the Blosum62 model of amino acid substitution, with bootstrapping of 100 replicates. The Molecular Evolutionary Genetics Analysis (version 6) tool MEGA6 (Tamura et al., 2013) was used to view and construct the phylogenetic tree. Adobe Illustrator CS5 and Microsoft Office Power Point were used to produce the final presentation of the tree.

### RNA Extraction and cDNA Synthesis

*Saprolegnia parasitica* strain “Coker 1923” (CBS 223.65; GenBank JX418013) was obtained from the Centraal Bureau voor Schimmel Culture (CBS, Baarn, Netherlands). Cultures were maintained on solid (2% agar) minimal medium (Machlis, 1953) at 25°C in the dark. For mycelium production, 5-mm plugs were excised from cultures on agar plates and incubated in liquid minimal medium (Machlis, 1953) without agitation for 3 days at 25°C in the dark. The resulting mycelium was washed three times with filter-sterilized water and frozen in liquid nitrogen for storage at -80°C. Total RNA was isolated from ground *S. parasitica* mycelium using the RNeasy Plant Mini Kit from Qiagen AB (Sollentuna, Sweden), used according to the manufacturer’s instructions, including treatment with RNase-free DNase to remove DNA contamination (Ambion TURBO DNA-free Kit from Thermo Fisher Scientific, Stockholm, Sweden). Template RNA for cDNA synthesis was first quantified using a NanoDrop 1000 spectrophotometer (Thermo Fisher Scientific), and the quality confirmed by gel electrophoresis. The Maxima First Strand cDNA Synthesis Kit was used to perform reverse transcription of the total RNA (Thermo Fisher Scientific).

### Cloning and Site-Directed Mutagenesis of SPRG_11783 and SPRG_17895

Preliminary sequence analysis of the proteins encoded by SPRG_11783 and SPRG_17895 revealed the absence of signal peptides in both proteins (SignalP tool^2^, but the presence of two potential transmembrane domains in the N-terminal region of SPRG_11783 (ExPASy TMPred tool ^3^).

Cloning primers for the SPRG_11783 and SPRG_17895 genes were designed using Primer 3Plus^4^. Primer sequences are listed in Supplementary Table S2. The full length amplicons of SPRG_11783 (2415 bp) and SPRG_17895 (1416 bp) were amplified from cDNA using a Phusion high fidelity polymerase and buffer system (Thermo Fisher Scientific). The PCR protocol utilized consisted of the following sequence: 98°C, 30 s; 35 cycles of [98°C, 10 s; 71 C, 25 s; 72°C, 25 s]; and 72°C, 5 min. The PCR products were purified from bands excised from a 1% agarose gel (Thermo Fisher Scientific GeneJET PCR Purification Kit) using a GeneRuler 1 kb DNA ladder to indicate that bands were of the correct size (Thermo Fisher Scientific). The Gateway^®^ System Ready was used for cloning, with the pENTR^TM^/SD/D-TOPO^®^ Vector as entry vector and the Gateway pET-DEST42 Vector as expression vector (both from Invitrogen, Carlsbad, CA, USA). The Gateway^®^ System was used according to the manufacturer’s instructions. The resulting expression plasmids included a gene for ampicillin resistance and a C-terminal hexahistidine (His_6_) tag for protein purification. Confirmation of the insertion and frame of the cloned genes was achieved by plasmid sequencing (Eurofins Genomics, Ebersberg, Germany). Plasmids were transformed into *Escherichia coli* cells by heat shock at 42 C for 30 s. OneShot *E. coli* Top10 cells (Thermo Fisher Scientific) were used for plasmid amplification. For protein production, plasmids were recovered using the Thermo Fisher Scientific GeneJET Plasmid Miniprep kit and were transformed into *E. coli* BL21 (DE3) cells (New England BioLabs Inc., Ipswich, MA, USA).

Point mutations were introduced into the SPRG_11783 gene by site-directed mutagenesis of the plasmid containing the gene, by a simple PCR-based method using the Pfx polymerase enzyme and buffer system (Thermo Fisher Scientific). Primers were used to introduce a N518H and/or a V522I mutation, altering the Y,N,V amino acid triad to Y,H,I; Y,H,V; or Y,N,I. Primer sequences are presented in Supplementary Table S2. The PCR protocol utilized in each case consisted of the following steps: 94°C, 5 min; 22 cycles of [94°C, 30 s; 55°C, 1 min; 68°C, 6 min]; 68°C, 15 min. The restriction enzyme *Dpn*I (Thermo Fisher Scientific) was used to degrade methylated parental DNA by adding 1 μL of the enzyme solution to 50 μL PCR reaction, and incubating for 1 h at 37 C. PCR product was purified using a PCR Clean-Up kit (Qiagen AB, Sollentuna, Sweden), and mutated plasmids were transformed into OneShot *E. coli* Top10 cells by heat shock. Plasmid sequences were confirmed to contain the desired mutation(s) by sequencing (Eurofins Genomics, Ebersberg, Germany). For protein production, constructs were transformed into *E. coli* BL21 cells.

### Protein Expression and Purification

One-liter cultures of BL21 DE3 *E. coli* cells harboring the desired plasmid were grown at 37°C with rotary shaking (180 rpm) in LB medium containing ampicillin (50 μg mL^-1^), to an OD600 of 0.4–0.6, when protein production was induced by the addition of IPTG (isopropyl α-D-thiogalactopyranoside) to a final concentration of 0.2 mM. The incubator temperature was lowered to 20°C, and protein production continued for 48 h. Cells were harvested by centrifugation at 5000 × *g* for 30 min, and were then re-suspended in 20 mL Buffer A (50 mM sodium phosphate buffer (pH 7.4) with 20 mM imidazole). Cells in buffer A were sonicated for 2 min to lyse cells. Proteins were separated from cell debris by centrifugation at 17000 × *g* for 30 min at 4°C. The supernatant liquid contained the over-expressed protein, which was His-tag purified by IMAC (immobilized metal ion affinity chromatography). Econo-Pac Chromatography Columns (Bio-Rad, Hercules, CA, USA) were used and loaded with 5 mL IMAC Sepharose^TM^ 6 Fast Flow resin (GE Healthcare, Uppsala, Sweden), charged with NiCl_2_ and pre-conditioned with Buffer A. Proteins were loaded onto the column and washed with four volumes of Buffer A to remove loosely bound non-specific proteins. His-tagged proteins were eluted using a step-wise gradient of 0–100% Buffer B (50 mM sodium phosphate buffer (pH 7.4) with 500 mM imidazole). Eluted proteins were concentrated and washed into 50 mM sodium phosphate pH 7.4 using a 30-kDa cut-off Amicon Ultra centrifugal filters (Merck Millipore, Cork, Ireland). Protein purity and approximate size were confirmed by SDS-PAGE, using a PageRuler Plus pre-stained protein ladder (Thermo Fisher Scientific). The concentration of purified SPRG_11783 protein was determined using the Bradford assay (Bradford, 1976). A band corresponding to the over-expressed protein was excised from an SDS-PAGE gel and subjected to in-gel trypsin proteolysis and LC-MS/MS analysis following the protocol described in Srivastava et al. (2013).

## Enzyme Activity Assays

### *In vivo* Assay

Ten milliliter cultures of BL21 *E. coli* cells transformed with plasmid DNA containing the gene of interest were grown at 37°C with rotary shaking (180 rpm) in LB medium containing ampicillin (50 μg mL^-1^) and additionally supplemented with 5 μL of a 5 nM stock OS (Sigma-Aldrich Sweden AB, Stockholm, Sweden, product number 41043), for a total amount of 10.7 μg OS added to the cultures. The OS was first solubilized in methanol, and the appropriate volume of this was pipette directly into the culture medium, with no additional compounds. When an OD600 of 0.4–0.6 was reached, recombinant protein production was induced by the addition of IPTG to a final concentration of 0.2 mM. Protein production was allowed to proceed overnight at 37°C. The cultures were then sonicated and sterols were extracted as outlined below. Control experiments were performed on non-induced cultures and induced cultures lacking OS. Additional control experiments using cells expressing green fluorescent protein instead of SPRG_11783 were also performed.

### *In vitro* Assay

2,3-Oxidosqualene (10.7 μg) was mixed with increasing amounts of the recombinant *Sp*LASA protein (37.5, 75, or 112.5 μg) purified as described above. The OS was first solubilized in methanol, and the appropriate volume of this was pipette directly into the reaction mixture, with no additional compounds. The assay was performed in 50 mM sodium phosphate buffer (pH 7.4) with a final volume of 1.5 mL and an incubation time of 24 h at 25°C. Cholesterol was used as an internal standard and added to the reaction mixtures prior to sterol extraction and analysis.

### Extraction and GC-MS Analysis of Sterols

A mixture of chloroform:methanol (1:2; 3.75 volumes) was added to each *in vitro* reaction mixture. After 1 h incubation at room temperature under continuous agitation the chloroform phase was recovered by centrifugation at 2000 × *g* and 17°C for 10 min. The solvent was evaporated under nitrogen gas and the dried sterols were silylated at 60°C for 1 h in the presence of a commercial mixture of BSTFA [*N,O*-bis(trimethylsilyl) trifluoroacetamide] and TMCS (trimethylchlorosilane) (99:1; Supelco, Sigma-Aldrich Sweden AB, Stockholm, Sweden) diluted in pyridine (1/1 v/v). The modified sterols were then dried under nitrogen gas and dissolved in hexane. Analysis of the sterol derivatives was performed by gas chromatography coupled to mass spectrometry [Hewlett-Packard, model 6890 (GC) and 5973 (MS)] using a CP-Sil 5 CB column (30 m × 0.25 mm; Agilent Technologies). Helium was used as a carrier gas (1 mL min^-1^). The temperature of the GC oven was initially set at 245°C, and successively increased to 265°C at a rate of 3.5°C min^-1^ and 310°C at a rate of 0.5°C min^-1^. MS spectra were recorded between 40 and 800 m/z. The identity of the detected sterols was confirmed by comparison to the GC retention times and MS fragmentation patterns of commercial standards run in this experiment and available in the literature. The peak area of the internal standard (cholesterol) was used to quantify the sterols present in the samples as well as OS.

## Results and Discussion

### Oxidosqualene Cyclases in *S. parasitica*

**FIGURE 2:**
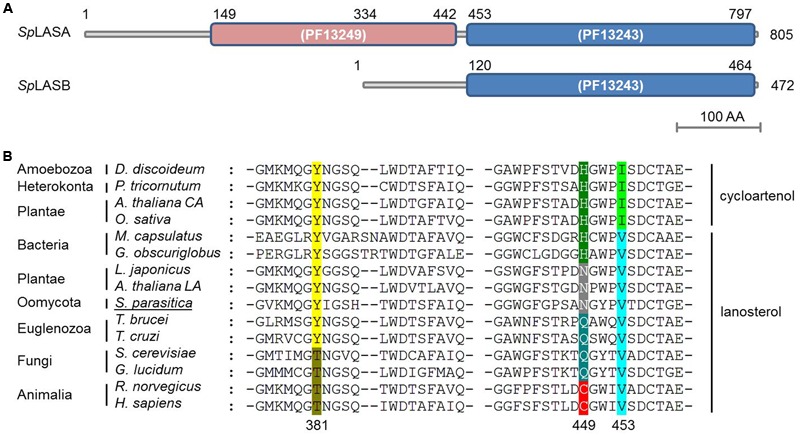
**Predicted domains of the two OSC enzymes from *S. parasitica* (*Sp*LASA and *Sp*LASB) and sequence alignment with similar enzymes from different taxa. (A)** *Sp*LASA (SPRG_11783) contains two separate Pfam squalene-hopene cyclase domains. *Sp*LASB (SPRG_17895) contains the C-terminal domain only. **(B)** Protein sequence alignment of diverse OSC enzymes highlighting a key amino acid triad involved in enzyme. The numbers given for the positions of the three amino acids are from the human OSC. Variations in these positions are highlighted with different colors and the enzymes are grouped into LAS and CAS. The *S. parasitica* sequence shown is from *Sp*LASA. *Arabidopsis thaliana* CA and *A. thaliana* LA refer to the CAS and LAS enzymes from *A. thaliana*, respectively.

To date, support for a reconstructed sterol synthesis pathway in *S. parasitica* has derived from bioinformatics and analysis of sterol profiles, showing that LA is abundant in the mycelium while CA is not detectable (Jiang et al., 2013; Warrilow et al., 2014). The putative OSC enzymes encoded by the genes SPRG_11783 and SPRG_17895 are proposed to perform the cyclisation of OS to LA, the first committed step of sterol biosynthesis in *S. parasitica* (Jiang et al., 2013). However, no functional characterization of these or any orthologous oomycete genes have yet been presented. SPRG_17895 encodes a 472 amino acid protein, which is a truncated duplicate of SPRG_11783, beginning at residue 334 of the full-length 805 amino acid sequence (Warrilow et al., 2014). The proteins encoded by SPRG_11783 and SPRG_17895 are hereafter referred to as *Sp*LASA and *Sp*LASB, respectively. Although *Sp*LASB is a significantly shorter protein, over the region shared by both proteins they show 99% identity. There are just three amino acid differences: compared to *Sp*LASA, *Sp*LASB has M470I, N492S, and A692V (positions numbered for *Sp*LASA). The predicted catalytic domain of *Sp*LASA contains two separate Pfam squalene-hopene cyclase domains (PF13249 at the N-terminal and PF13243 at the C-terminal) (Finn et al., 2016) (**Figure 2A**). *Sp*LASB contains solely the C-terminal domain, so it is unclear whether this protein is catalytically active. Further sequence analysis of *Sp*LASA indicates the presence of two potential transmembrane domains at the N-terminal end of the protein (amino acids 19–39 and 164–183), which are not found in *Sp*LASB. Due to the very high level of sequence similarity between the two genes, it is difficult to determine whether both are expressed by *S. parasitica.* Our own preliminary analyses (qPCR experiments, data not shown) indicate that SPRG_11783 is expressed in *S. parasitica* mycelium. Additionally, evidence was presented with the sequencing of the *S. parasitica* genome that the SPRG_17895 gene is expressed, and is more highly expressed in cysts than in the mycelium (Jiang et al., 2013).

Sequence alignment of *Sp*LASA and *Sp*LASB with other known and predicted OSCs showed that both oomycete proteins contain an unusual Y,N,V pattern at amino acid positions 450, 518, and 522 for *Sp*LASA, and 117, 185, and 199 for *Sp*LASB (**Figure 2B**). Previous experiments have demonstrated that these positions are crucial in distinguishing LAS and CAS enzymes (Summons et al., 2006), although the precise role of these amino acids in activity, substrate binding, or other mechanistic action, is unknown. This triad of amino acids is more variable in LAS than CAS enzymes, where it appears to always be Y,H,I (**Figure 2B**). The Y,N,V pattern of the *S. parasitica* proteins has previously only been observed in plant enzymes, characterized as LAS in *A. thaliana* and *Lotus japonicus* (Kolesnikova et al., 2006; Suzuki et al., 2006) (**Figure 2B**).

### Phylogenetic Analysis of OSC Enzymes

**FIGURE 3:**
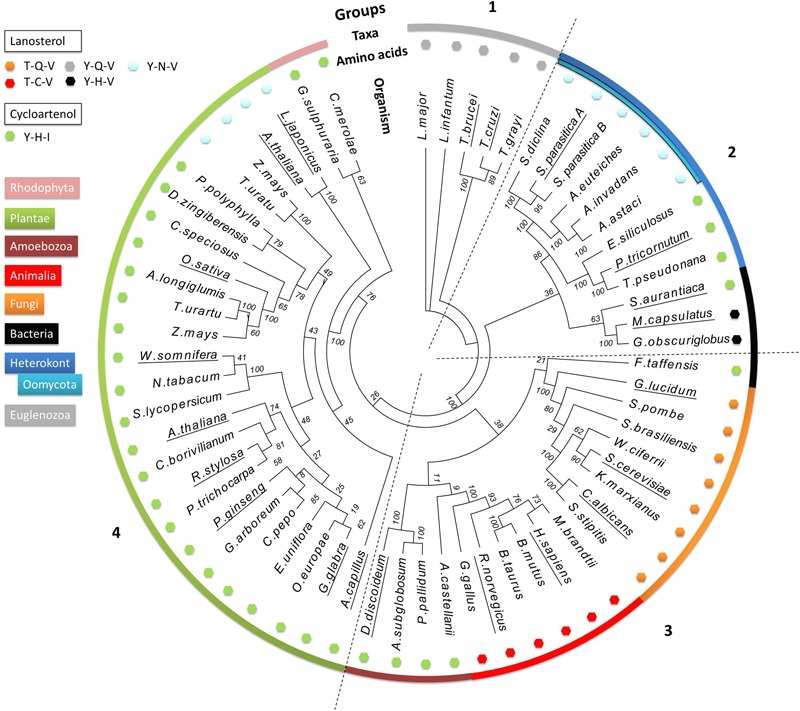
**Phylogenetic analysis of 63 characterized and predicted OSC sequences.** Species group into four clades, numbered 1–4. The outer colored ring denotes the taxonomic groups of the species from which the sequences originate, as indicated by the color key. The small colored hexagons each correspond to a different triad amino acid pattern. The names of the species for which enzyme specificity has been biochemically demonstrated are underlined. Bootstrap values from 100 replicate analyses are shown at each root branch. Full organism names and sequence identification numbers are listed in Supplementary Table S1.

We performed a phylogenetic analysis of LAS and CAS enzymes using 63 characterized or predicted OSC protein sequences from different taxonomic groups of organisms (Supplementary Table S1). A phylogenetic tree was produced using only the predicted catalytic domains of the selected sequences to exclude interference from non-catalytic domains. The tree allows comparison of the occurrence of different amino acid motifs, enzyme specificities, and sterol profiles. This analysis is unlikely to be sufficient to infer a complete evolutionary history of OSC genes. Nevertheless, four distinct clades are apparent (**Figure 3**). Group 1 contains only sequences from the Euglenozoa phylum. Group 2 contains sequences from the Heterokonta phylum, including the oomycete class, as well as three bacterial sequences. Group 3 includes sequences from animals, fungi, amoeba, and one bacterium. Group 4 contains enzymes from plants and red algae. The bacteria are the only taxonomic group not all found in the same clade: they are found in Groups 2 and 3. All predicted CAS enzymes have the Y,H,V motif, whereas there are five different amino acid patterns in predicted LAS enzymes ([T,C,V]; [T,Q,V]; [Y,Q,V]; [Y,H,V]; [Y,N,V]). Groups 2–4 all contain examples of both CAS and LAS sequences. Some plant species have both LAS and CAS genes, but so far it has been conclusively demonstrated only for *A. thaliana* that both are functional (Ohyama et al., 2009).

Sequences from the Euglenozoa (Group 1) are distinct from all other OSC enzymes, and possess a unique Y,Q,V amino acid pattern. Examples from *Trypanosoma brucei* and *Trypanosoma cruzi* have been biochemically characterized as LAS, and therefore we hypothesize that the others in the group share the same activity (Buckner et al., 2000; Joubert et al., 2001).

The next branching point clearly separates the heterokonts, including the oomycetes, from the other clades. Group 2 contains both CAS and LAS enzymes. The CAS enzymes from the algae *Ectocarpus siliculosus, Thalassiosira pseudonana*, and *Phaeodactylum tricornutum* [experimentally shown to increase CA accumulation in transformed yeast (Fabris et al., 2014)] have the classical Y,H,I motif, while the oomycete LAS enzymes all have the Y,N,V motif seen in *Sp*LASA and *Sp*LASB. We identified predicted LAS sequences from the oomycetes *Saprolegnia diclina, Aphanomyces euteiches, Aphanomyces invadans*, and *Aphanomyces astaci* (**Figure 3**). The same Y,N,V motif is also present in some plant enzymes (Group 4, see below). A sub-group containing three bacterial OSC sequences also clusters in Group 2 (*Stigmatella aurantiaca, Methylococcus capsulatus* and *Gemmata obscuriglobus*). These display both CAS and LAS amino acid patterns, including the LAS Y,H,V triad, which appears to be unique to bacteria. Y,H,V bacterial sequences have been proposed as representing an early ancestral OSC gene, suggesting that the earliest sterol synthesis pathways originated in prokaryotes and proceeded via LA production (Pearson et al., 2003; Lamb et al., 2007; Nakano et al., 2007). The oomycete sequences may derive from this early ancestor, after a single amino acid change to Y,N,V. One could perhaps speculate that the algal sequences may derive from an early Y,H,I bacterial ancestor (e.g., *S. aurantiaca*), but more investigation is needed to be sure of this.

Group 3 sequences are all predicted LAS enzymes, with the exception of a sub-group of amoebal enzymes and the sole bacterial sequence in the group (*Fluviicola taffensis*). The amoebal and bacterial sequences possess the Y,H,I motif and are predicted to be CAS. This motif differs significantly from the T,Q,V and T,C,V triads dominating the clade, with three separate changes in the amino acid patterns, implying multiple mutational events between sequences otherwise similar enough to co-cluster in this analysis. Some bacterial OSC genes may have been obtained via horizontal transfer event(s) from other organisms (Desmond and Gribaldo, 2009), perhaps explaining the existence of an OSC in *F. traffensis* so dissimilar to the bacterial sequences in Group 2 (**Figure 3**).

Group 4 mostly comprises CAS enzymes, with the exception of a sub-group of unusual plant LAS enzymes, which have a Y,N,V motif, a two amino acid difference from the CAS Y,H,I motif in the rest of the clade. As the Group 4 LAS sequences have the same triad as the oomycete enzymes, it is tempting to suggest some cross-species transfer of genetic information leading to this dual appearance of the motif in such distinct clades. However, although the monocot (*Zea mays* and *Triticum urartu*) and dicot (*L. japonicus* and *A. thaliana*) LAS genes share the Y,N,V amino acid pattern, they cluster quite distinctly from each other on the tree. The dicot LAS genes therefore appear to have emerged from an earlier ancestor than the monocot LAS genes (**Figure 3**). This finding is consistent with previous suggestions that the synthesis of sterols in dicot plants derive from an ancestral LAS (Xue et al., 2012). To date, no monocot LAS enzymes have been shown to support a functional LA synthesis pathway in plants (Xue et al., 2012). The monocot LAS sequences in our analysis grouped with plant CAS genes, which conforms to a previously proposed theory that monocot LAS genes derive from a duplicated CAS gene (Xue et al., 2012). A similar gene duplication event may explain the presence of two apparent OSC genes in the *S. parasitica* genome, encoding *Sp*LASA and *Sp*LASB.

If the Y,H,V motif is indeed a feature of a common ancestral bacterial LAS, from which OSCs in other species diverged (Pearson et al., 2003; Lamb et al., 2007), we can hypothesize that three distinct single mutations have occurred, leading separately to the Y,Q,V (Euglenazoa LAS), Y,N,V (oomycete LAS), and Y,H,I (CAS) sequences. Higher order eukaryotes with the T,Q,V (fungi) or T,C,V (animals) motif in their OSC enzymes have undergone two changes from the ancestral sequence. The Y381T (position numbered for the human LAS) change may have arisen in an ancestor common to animals and fungi. The animal and fungal proteins then separately underwent an additional change from T,H,V, leading to different sequences with the same LAS specificity.

### Functional Characterization of *Sp*LASA

#### *Sp*LAS Cloning, Expression, and Purification

**FIGURE 4:**
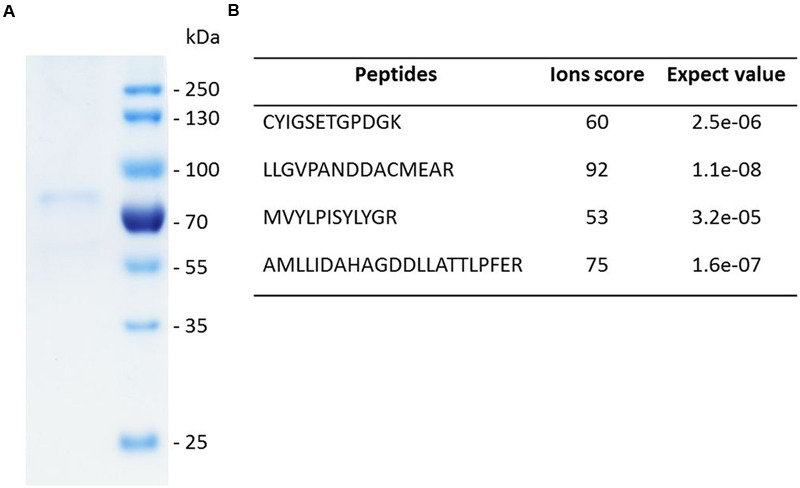
**SDS-PAGE analysis and identity confirmation of the recombinant *Sp*LASA protein. (A)** The recombinant protein expressed in *E. coli* was purified by immobilized metal ion affinity chromatography (IMAC). **(B)** The identity of the Coomassie blue-stained SDS-PAGE band was confirmed by mass spectrometry analysis after in-gel partial proteolysis with trypsin. The peptides identified unequivocally confirmed that the purified recombinant protein corresponds to *Sp*LASA. The corresponding spectra are presented in Supplementary Figure S2.

The genes encoding *Sp*LASA and *Sp*LASB were amplified from cDNA by PCR. They were then cloned into the pET-DEST42 Vector which contains a C-terminal His_6_ tag for affinity purification of recombinant proteins. Site-directed mutagenesis of *Sp*LASA was used to produce changes in the Y,N,V amino acid triad at positions 450, 518, and 522, to make [Y,H,V]; [Y,N,I]; and [Y,H,I]. Following sequence confirmation, plasmids of *Sp*LASA, *Sp*LASB, and the mutant variants of *Sp*LASA, were transformed into *Escherichia coli* strain BL21 (DE3) for over-expression. Production and purification of soluble protein was successful in the case of *Sp*LASA. This protein was purified by two rounds of IMAC, and its successful enrichment was demonstrated by SDS-PAGE analysis (**Figure 4A**), which also confirmed the purified protein has the predicted molecular weight of ∼91 kDa. Additional silver staining of a gel revealed the presence of some faint protein bands, likely arising from the bacterial expression strain (Supplementary Figure S1): several control experiments were performed to ensure that no OSC activity could be linked to these bacterial proteins (see below). Identification of the purified recombinant protein was additionally confirmed by mass spectrometric analysis of peptides generated by trypsin hydrolysis of an excised gel band (**Figure 4B**; Supplementary Figure S2). *Sp*LASA expression yields were low, with typically around 50–100 μg purified protein recoverable from one liter bacterial culture. This is in accordance with previous reports describing the difficulty of OSC production in bacterial expression strains (Corey et al., 1997), and may be due to the predicted transmembrane regions of *Sp*LASA, which rendered the protein unstable in the intracellular bacterial environment. However, *Sp*LASA yields in our experiments were consistent and reproducible, and bacterial cultivation could be scaled up to produce sufficient protein for enzymatic assays. Despite repeated attempts and large volumes of culture, no over-expression of *Sp*LASB, or any mutant variant of *Sp*LASA could be achieved. No bands were visible on SDS-PAGE by Coomassie stains, but protein samples were still subjected to trypsin proteolysis and MS analysis to screen for low levels of these recombinant proteins: no peptides corresponding to *S. parasitica* proteins were detected in any case. Cultures of cells transformed with these plasmid also showed no activity in the *in vivo* assay (see below).

#### *In vivo* Conversion of OS

**FIGURE 5:**
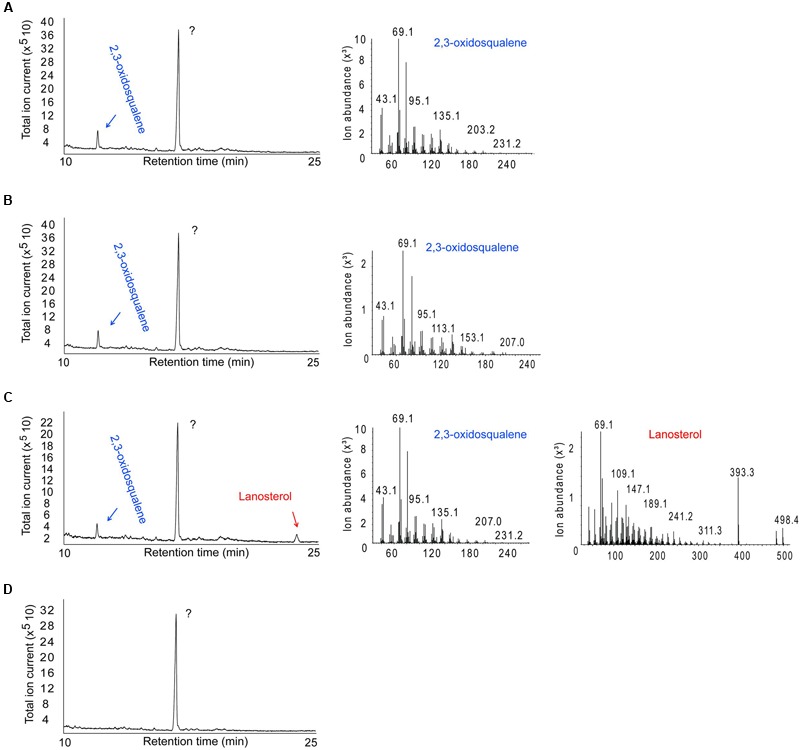
**GC-MS analysis of the product formed by *E. coli* cells expressing *Sp*LASA and grown in the presence of the OS substrate. (A)** Control *E. coli* BL21 cells producing green fluorescent protein showed no conversion of the OS substrate. **(B)** *E. coli* cells that expressed *Sp*LASA but that were not induced by IPTG for protein expression showed no conversion of OS. **(C)** *E. coli* cells that expressed *Sp*LASA and were induced by IPTG for protein expression specifically converted a portion of the OS substrate to LA. **(D)** Negative control performed on *E. coli* cells producing *Sp*LASA and grown in the absence of the OS substrate shows no conversion of OS. The major peak detected in all samples is an unidentified compound that does not correspond to any sterol as judged by MS analysis (not shown). The identity of the OS and LA GC peaks were assigned by comparison to standards and confirmed by inspection of their MS fragmentation patterns.

*Escherichia coli* BL21 cells harboring the SPRG_11783 gene in cultures supplemented with OS were assessed for their ability to cyclize the molecule into a sterol. Control experiments were performed using cells transformed with a plasmid with a non-OSC gene (green fluorescent protein, GFP). Following IPTG induction for protein expression, and incubation for a further 16 h, *E. coli* cells were sonicated in the growth medium, followed by chloroform-methanol extraction of sterols. Analysis by GC-MS confirmed the presence of LA in the cultures producing *Sp*LASA (**Figure 5**). Furthermore, LA was not detected in the control bacterial cultures carrying the GFP gene, or in *Sp*LASA cultures which were not induced, or in induced *Sp*LASA cultures not supplemented with OS (**Figure 5**). These data confirm unequivocally that *Sp*LASA has LAS activity, conferring upon the host bacterial cells the ability to convert OS into LA. No other sterols were detected in the *Sp*LASA samples, showing that LA is the only catalytic reaction product under these conditions. The stability of OS in LB medium was assessed by incubating 10.7 μg of the substrate in 1.5 mL medium overnight at room temperature. GC-MS analysis showed that there was no spontaneous conversion of the substrate into LA (Supplementary Figure S3). We hypothesize that the conversion of OS into LA by the cells over-expressing *Sp*LASA occurred after uptake of the substrate into the cells. A detectable amount of conversion only took place when the OS was included in culture media from the beginning of bacterial growth. Adding the OS after the induction of protein expression did not lead to conversion to LA. Likewise, adding the OS to sonicated cells and incubating for a 24 h period resulted in no conversion to LA.

#### *In vitro* Conversion of OS by *Sp*LASA

**FIGURE 6:**
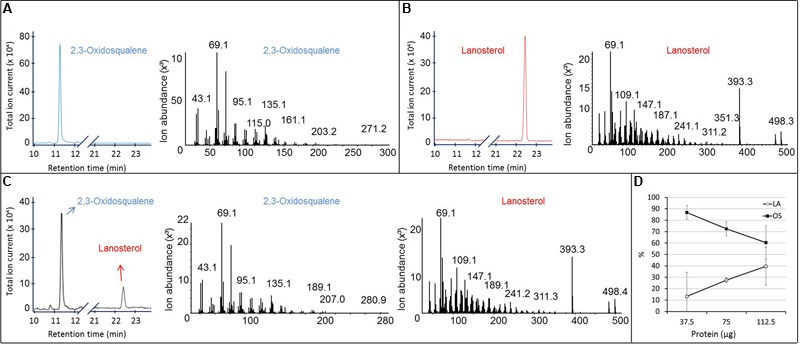
**GC-MS analysis of the product formed *in vitro* by the purified recombinant *Sp*LASA protein expressed in *E. coli*. (A,B)** Gas chromatogram and corresponding MS fragmentation pattern of OS and LA standards, respectively. **(C)** GC-MS analysis of the reaction mixture recovered after incubation of 112.5 μg of the purified recombinant *Sp*LASA protein with 10.3 μg OS showing conversion of OS to LA. **(D)** Incubation of increasing amounts of the purified recombinant *Sp*LASA with a constant amount of OS (10.3 μg). The amount of LA formed increased linearly in the presence of increasing amounts of *Sp*LASA. Reciprocally, the amount of OS recovered at the end of the reaction decreased linearly.

Purified recombinant *Sp*LASA was tested for its ability to cyclize OS by incubating 10.7 μg of OS with 37.5, 75, or 112.5 μg of the protein for 24 h at 25°C. Sterols were extracted from the reaction mixtures, silylated, and analyzed by GC-MS (**Figure 6**). These experiments confirmed the specific conversion of OS into LA. No other cyclized products were identified, whereas traces of several reaction products have been detectable in assays of OSC enzymes from *A. thaliana* and other organisms (Phillips et al., 2006). Quantification of substrate and product by GC-MS analysis after enzymatic reactions showed that the proportion of LA increased linearly with increasing protein concentration, while the OS concentration decreased proportionally (**Figure 6D**). This dose-dependency of the reaction confirms that the cyclisation is enzymatically catalyzed, as increasing protein concentration led to an increased conversion of substrate into product. Additionally, our preliminary stability tests showed that there is no spontaneous cyclisation of the substrate in water or the reaction buffer (Supplementary Figure S3). The total amount of material recovered by chloroform-methanol extraction remained relatively constant in all samples (Supplementary Figure S4), supporting the accuracy of these quantification values.

#### Implications for Oomycete Sterol Synthesis

The inhibition of sterol synthesis in the oomycetes is a promising avenue for targeted applications in the fight against the spread of these pathogenic microorganisms. The drug clotrimazole, which inhibits a CYP51 enzyme involved in sterol synthesis, has been shown to be effective at controlling the growth of *S. parasitica* (Warrilow et al., 2014), and other steps in the biosynthetic pathway are potential targets for similar disruption. *Sp*LASA is the first biochemically characterized oomycete OSC and may represent a drug target for sterol synthesis inhibition. Indeed, the use of OSC inhibitors as anti-microbial agents has been successfully explored for some human pathogens (Buckner et al., 2001; Hinshaw et al., 2003). However, CYP51 inhibitors tend to be broadly acting, and may affect not only the oomycete pathogen but also animals and plants in the surrounding local environment. Conversely, a highly targeted inhibition of OSCs with the Y,N,V amino acid triad would enable precise disruption of sterol synthesis in oomycete pathogens. Design and application of a drug with such a high level of specificity will require much more detailed knowledge of the structure and mechanism of oomycete LAS enzymes. With structural and mechanistic information available it may become possible to develop new molecules that mimic transition state or intermediate compounds formed from OS during the cyclisation reaction (Buckner et al., 2001; Hinshaw et al., 2003). Targeted disruption of the SPRG_11783 gene would be necessary to confirm that *Sp*LASA is the only functional LAS in *S. parasitica*. It may be that the truncated protein *Sp*LASB, or another unidentified protein, can provide an alternative mode of sterol synthesis or acquisition. This would compensate for the loss of active *Sp*LASB. Such investigations may become possible in the near future, provided that an efficient method for gene silencing can be successfully applied to *S. parasitica*. The CRISPR/Cas9 approach recently used to disrupt an effector gene in the oomycete *Phytophthora sojae* (Fang and Tyler, 2016) holds promise for silencing genes in other oomycetes such as *S. parasitica* and perform *in vivo* functional characterization.

## Author Contributions

PD and VS performed the research, with input from LM and VB. LM and VB designed and supervised the research, and all authors contributed to data analysis and interpretation. LM and VB wrote the manuscript, with input from PD and VS.

## Conflict of Interest Statement

The authors declare that the research was conducted in the absence of any commercial or financial relationships that could be construed as a potential conflict of interest.

## Footnotes

1 http://blast.ncbi.nlm.nih.gov/Blast.cgi

2 http://www.cbs.dtu.dk/services/SignalP/

3 http://embnet.vital-it.ch/software/TMPRED_form.html

4 http://www.bioinformatics.nl/cgi-bin/primer3plus/primer3plus.cgi

## Abbreviations

CA: cycloartenol
CAS: cycloartenol synthase
LA: lanosterol
LAS: lanosterol synthase
OS: 2,3-oxidosqualene
OSC: oxidosqualene cyclase
