# Supplementary material for: The Oxidosqualene Cyclase from the Oomycete Saprolegnia parasitica Synthesizes Lanosterol as a Single Product
Source: Front Microbiol. 2016 Nov 9;7:1802. doi: 10.3389/fmicb.2016.01802 (PMC5101207; doi:10.3389/fmicb.2016.01802)

Supplementary Material

The Oxidosqualene Cyclase from the Oomycete *Saprolegnia Parasitica* Synthesizes Lanosterol as a Single Product

**Paul Dahlin, Vaibhav Srivastava, Vincent Bulone^*^, Lauren S. McKee^*^**

*** Correspondence:** bulone@kth.se; mckee@kth.se

**Table S1** **Putative oxidosqualene cyclase (OSC) sequences used to generate the phylogenetic tree presented in Fig. 3.** Sequences are identified by organism name, GI number and Accession number. Where a species is known to possess two OSC sequences, the predicted specificity of the enzyme is indicated by (LA) or (CA). For genes which have been functionally characterized, the appropriate literature reference is provided.

| [**Organism**](http://www.ncbi.nlm.nih.gov/Taxonomy/Browser/wwwtax.cgi?id=130081) | **Sequence identification numbers** | | **Reference if biochemically characterized** |
| --- | --- | --- | --- |
|  | **GI** | **Accession** |  |
| *Leishmania major* Friedlin | 157864248 | XP_001680835.1 |  |
| *Leishmania infantum* | 146071565 | XP_001463147.1 |  |
| *Trypanosoma brucei brucei* TREU927 | 72391704 | XP_846146.1 | Buckner *et al*. (2000) *Mol Biochem Parasitol* **110,** 399–403. |
| *Trypanosoma cruzi* CL Brener | 71668047 | XP_820967.1 | Joubert *et al.* (2001) *Org Lett* **14,** 1957–1960. |
| *Trypanosoma grayi* | 686635187 | XP_009310039.1 |  |
| *Saprolegnia diclina* VS20 | 669168079 | XP_008619537.1 |  |
| *Saprolegnia parasitica* (SPRG_11783) | 813184406 | XP_012206375 |  |
| *Saprolegnia parasitica* (SPRG_17895) | 813227241 | XP_012212697 |  |
| *Aphanomyces euteiches* | 189026977 | CAQ55984.1 |  |
| *Aphanomyces invadans* | 673053613 | XP_008880767.1 |  |
| *Aphanomyces astacis* | 698813722 | XP_009839890 |  |
| *Ectocarpus siliculosus* | 299115454 | CBN75619.1 |  |
| *Phaeodactylum tricornutum* CCAP 1055/1 | 209582527 | ACI65148.1 | Fabris *et al.* (2014) *New Phytol* **204,** 521–535. |
| *Thalassiosira pseudonana* CCMP1335 | 220976548 | EED94875.1 |  |
| *Stigmatella aurantiaca* DW4/3-1 | 309395729 | ADO73188.1 | Bode *et al.* (2003) *Mol Microbiol* **47,** 471–81. |
| *Methylococcus capsulatus* | 81680845 | Q603D4 | Lamb *et al.* (2007) *Mol Biol Evol* **24,** 1714–1721. Nakano *et al.* (2007) *Biosci Biotechnol Biochem* **71,** 2543–2550. |
| *Gemmata obscuriglobus* | 497722277 | WP_010036461.1 |  |
| *Fluviicola taffensis* | 327320695 | AEA45187.1 |  |
| *Ganoderma lucidum* | 290767253 | ADD60469.1 | Shang *et al.* (2010) *Biosci Biotechnol Biochem* **74,** 974–978. |
| *Schizosaccharomyces pombe* | 1706694 | Q10231.1 |  |
| *Sporothrix brasiliensis* | 748536820 | KIH87129.1 |  |
| *Wickerhamomyces ciferrii* | 406603243 | CCH45222.1 |  |
| *Saccharomyces cerevisiae* S288c | 341941150 | P38604.6 | Oliaro-Bosso *et al.* (2005) *ChemBioChem* **6,** 2221–2228. |
| *Kluyveromyces marxianus* | 693902145 | BAP71121.1 |  |
| *Candida albicans* P75010 | 723191577 | KHC65374.1 | Kelly, *et al.* (1990) *Gene* **87,** 177–183. |
| *Scheffersomyces stipitis* | 150865294 | XP_001384446.2 |  |
| *Myotis brandtii* | 521033784 | EPQ15569.1 |  |
| *Homo sapiens* | 1352387 | P48449.1 | Tian & Eriksson (2012) *J Phys Chem B* **116,** 13857–13862. |
| *Bos mutus* | 440900494 | ELR51621.1 |  |
| *Bos taurus* | 114053041 | NP_001040029.1 |  |
| *Rattus norvegicus* | 13591981 | NP_112311.1 | Abe & Prestwich (1995) *Lipids* **30,** 231–234. |
| *Gallus gallus* | 408358018 | NP_001006514.2 |  |
| *Acanthamoeba castellanii* Neff | 470371311 | XP_004332827.1 |  |
| *Polysphondylium pallidum* PN500 | 281207023 | EFA81207.1 |  |
| *Acytostelium subglobosum* | 831786716 | XP_012758197.1 |  |
| *Dictyostelium discoideum* | 74858627 | Q55D85.1 | Godzina *et al.* (2000) *Lipids* **35,** 249–255. |
| *Adiantum capillus-veneris* | 160081609 | BAF93208.1 | Shinozaki *et al.* (2008) *FEBS Lett* **582,** 310–318. |
| *Glycyrrhiza glabra* | 75266680 | BAA76902.1 | Hayashi *et al.* (2004) *Biol Pharm Bull* **27,** 1086–1092. |
| *Olea europaea* | 6456465 | BAA86931.1 |  |
| *Eugenia uniflora* | 672917058 | AIK19224.1 |  |
| *Cucurbita pepo* | 50896401 | BAD34644.1 |  |
| *Gossypium arboreum* | 728848450 | KHG27893.1 |  |
| *Panax ginseng* | 3688598 | BAA33460.1 | Kushiro *et al.* (1998) *Eur J Biochem* **256,** 238–244. |
| *Populus trichocarpa* | 566215895 | XP_006372242.1 |  |
| *Rhizophora stylosa* | 152962678 | BAF73929.1 | Oku *et al.* (2007) *Biosci Biotechnol Biochem* **71,** 1788–1792. |
| *Chlorophytum borivilianum* | 699263077 | AIU41742.1 |  |
| *Arabidopsis thaliana* (CA) | 21542399 | NP_178722.1. NM_126681.2. | Lodeiro *et al.* (2004) *ChemBioChem* **5,** 1581–1585. |
| *Solanum lycopersicum* | 168805621 | ACA28830.1 |  |
| *Nicotiana tabacum* | 725824123 | AIY33888.1 |  |
| *Withania somnifera* | 295919782 | ADG60271.1 | Dhar *et al.* (2014) *J Biol Chem* **289,** 17249–17267. |
| *Zea mays* (CA) | 226533427 | NP_001152006.1 |  |
| *Triticum urartu* (CA) | 474362672 | EMS63323.1 |  |
| *Avena longiglumis* | 47834383 | AAT38889.1 |  |
| *Oryza sativa* subsp. japonica | 937901856 | NP_001045848.1; BAS76910.1 | Ito *et al.* (2011) *Organic Lett* **13,** 2678–2681. |
| *Cheilocostus speciosus* | 18147771 | BAB83253.1 |  |
| *Dioscorea zingiberensis* | 145651385 | CAM91422.1 |  |
| *Paris polyphylla* var. yunnanensis | 406654340 | AFS49705.1 |  |
| *Triticum urartu* (LA) | 474193899 | EMS58271.1 |  |
| *Zea mays* (LA) | 226533427 | NP_001152006.1 |  |
| *Arabidopsis thaliana* (LA) | 145339142 | NP_190099.3 | Kolesnikova *et al.* (2006) *Arch Biochem Biophys* **447,** 87–95. |
| *Lotus japonicus* (LA) | 108743269 | BAE95410.1 | Sawai *et al.* (2006) *Plant Cell Physiol* **47,** 673–677. |
| *Galdieria sulphuraria* | 545701071 | XP_005702872.1 |  |
| *Cyanidioschyzon merolae* strain 10D | 544212095 | XP_005534767.1 |  |

**Table S2** **Primer sequences used in this study.**

| **Primer pair** | **Forward** | **Reverse** |
| --- | --- | --- |
| SPRG_11783 | CACCATGGCACGTGAAGCAC | GAGCGACTGGCCAGGTTGG |
| SPRG_17895 | CACCATGACGCCGTACAGTCAAGTG | GAGCGACTGGCCAGGTTGG |
| Y,N,I | GGACCGTCGGCCAACGGGTACCCAATTACGGACTGCACGGGCGAG | CTCGCCCGTGCAGTCCGTAATTGGGTACCCGTTGGCCGACGGTCC |
| Y,H,V | GGACCGTCGGCCCATGGGTACCCAGTCACGGACTGCACGGGCGAG | CTCGCCCGTGCAGTCCGTGACTGGGTACCCATGGGCCGACGGTCC |
| Y,H,I | GGACCGTCGGCCCATGGGTACCCAATTACGGACTGCACGGGCGAG | CTCGCCCGTGCAGTCCGTAATTGGGTACCCATGGGCCGACGGTCC |

**Figure S1.** Silver staining of an SDS-PAGE analysis of the recombinant *Sp*LASA protein. The protein was loaded at a high (H) and low (L) concentration. In the lane with higher protein loading, some faint bands are visible which could not be seen by Coomassie blue staining of the gel.


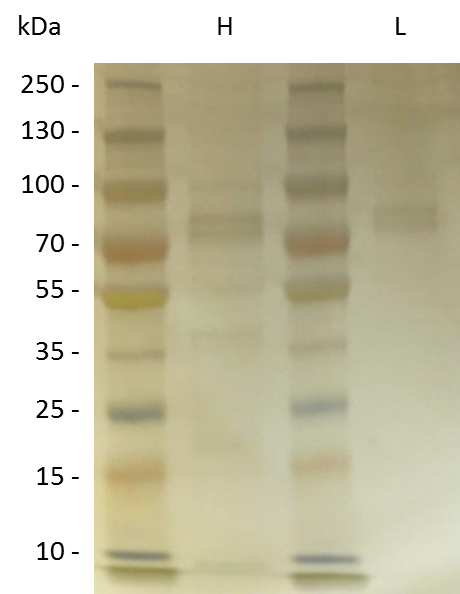


**Figure S2.** Mass Spectrometry analysis of the purified recombinant *Sp*LASA protein expressed in *E. coli*. Collision-induced dissociation (CID) spectra of peptides identified by mass spectrometry are presented. The detected b and y ions and the corresponding amino acid sequences are indicated together with the ion score and expect value for each peptide.

**CYIGSETGPDGK** (Ions score: 60, Expect value: 2.1e-06)
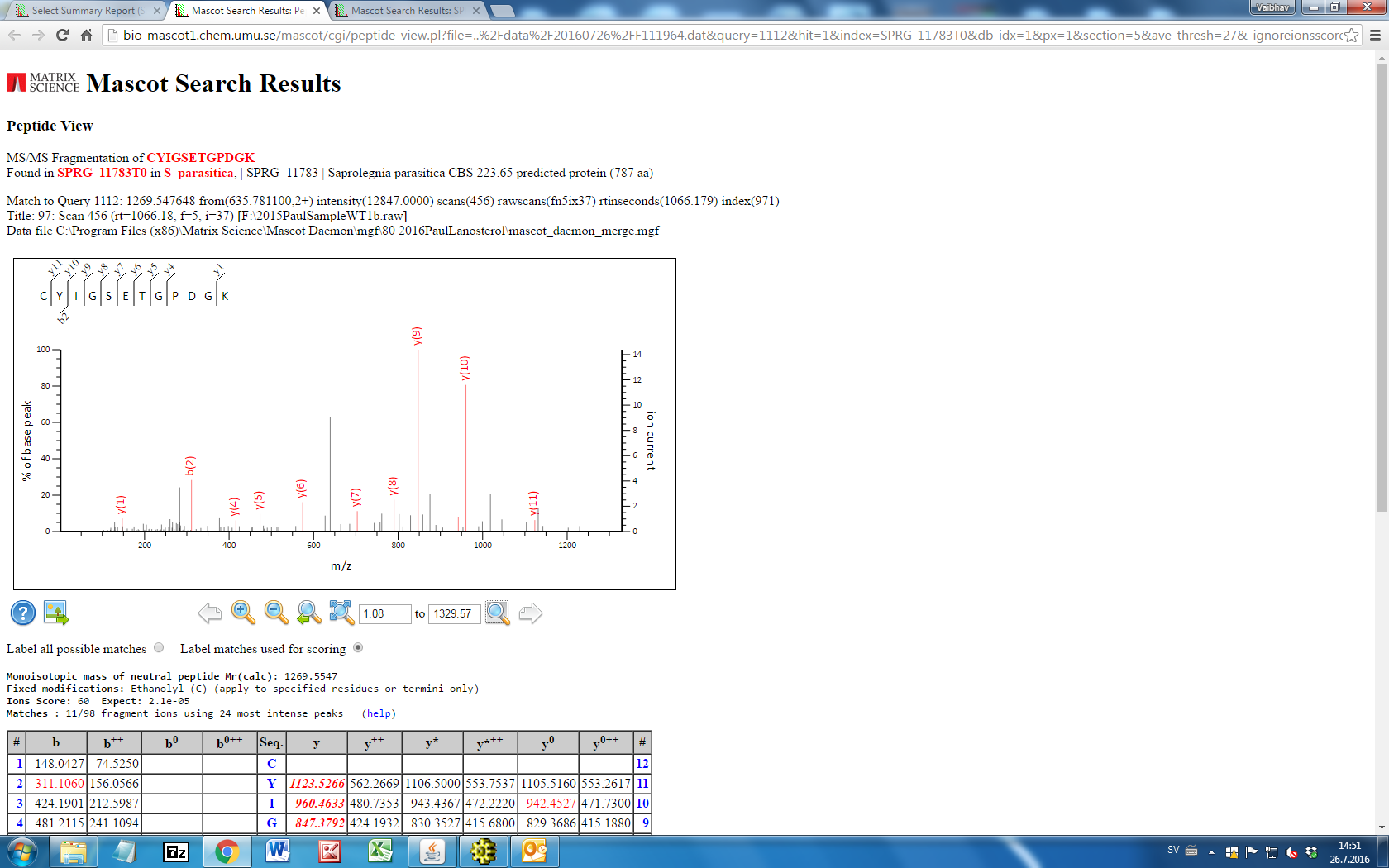


**LLGVPANDDACMEAR** (Ions score: 92, Expect value: 1.1e-08)
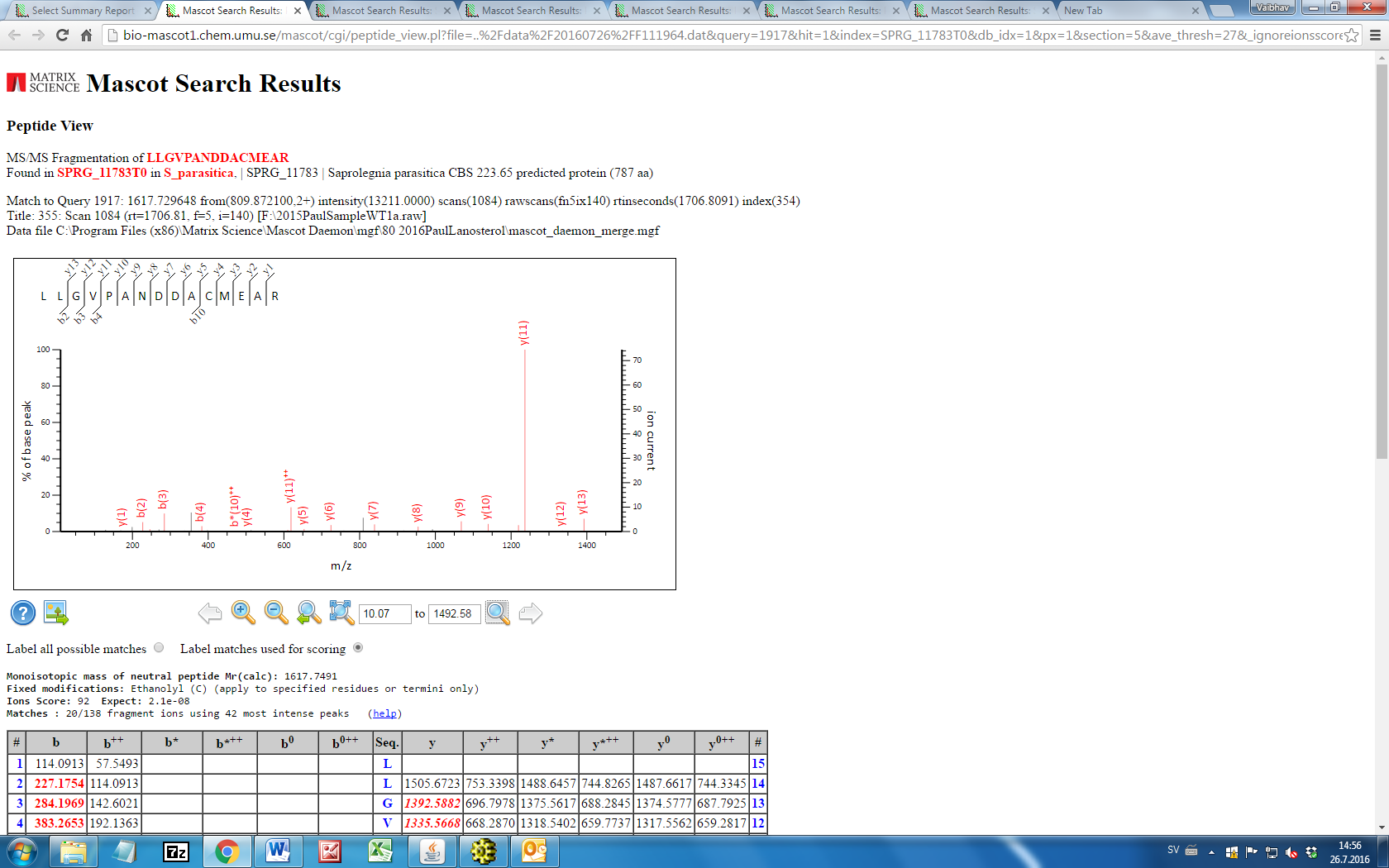


**MVYLPISYLYGR** (Ions score: 53, Expect value: 3.3e-05)
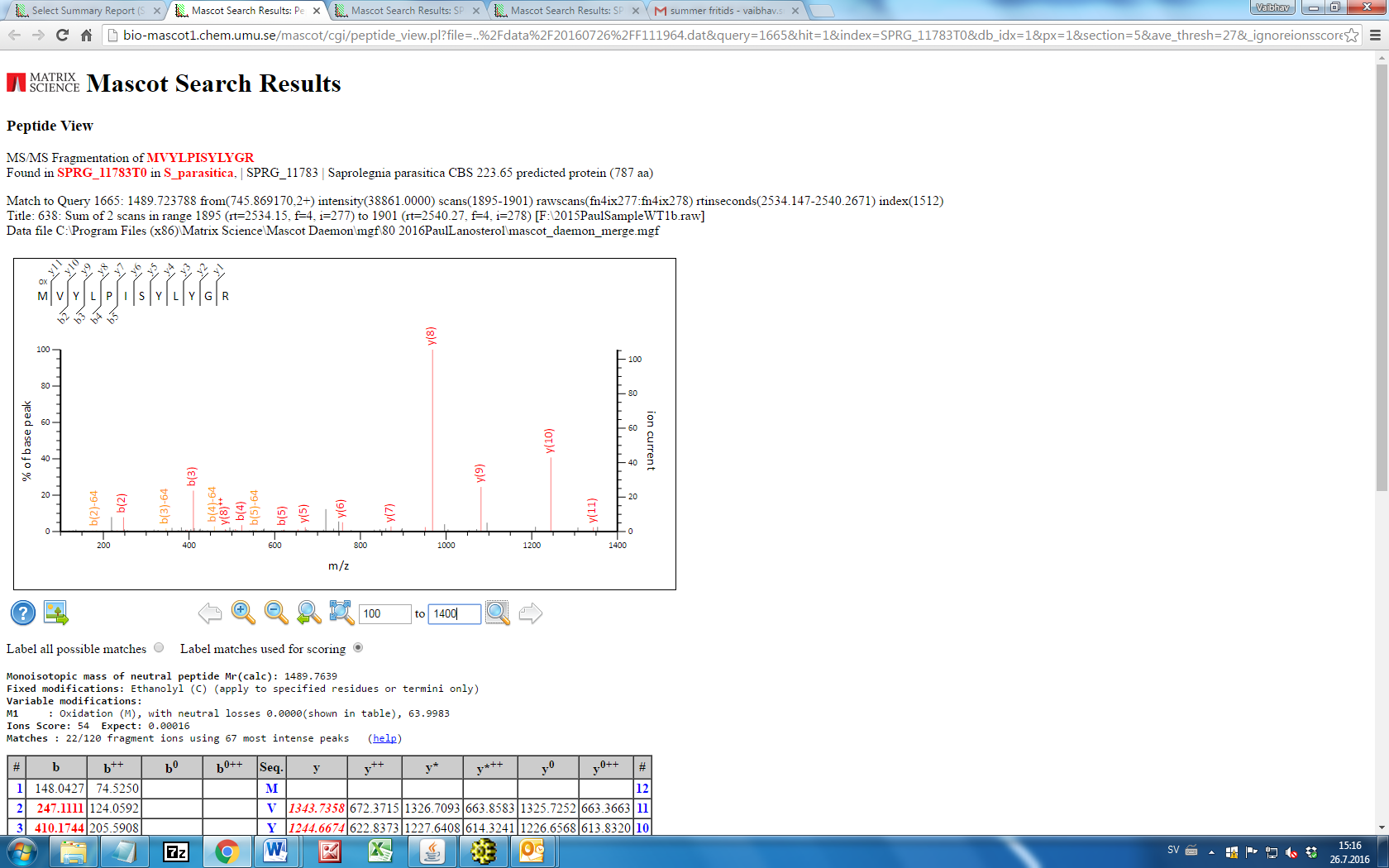


**AMLLIDAHAGDDLLATTLPFER** (Ions score: 75, Expect value: 1.6e-07)
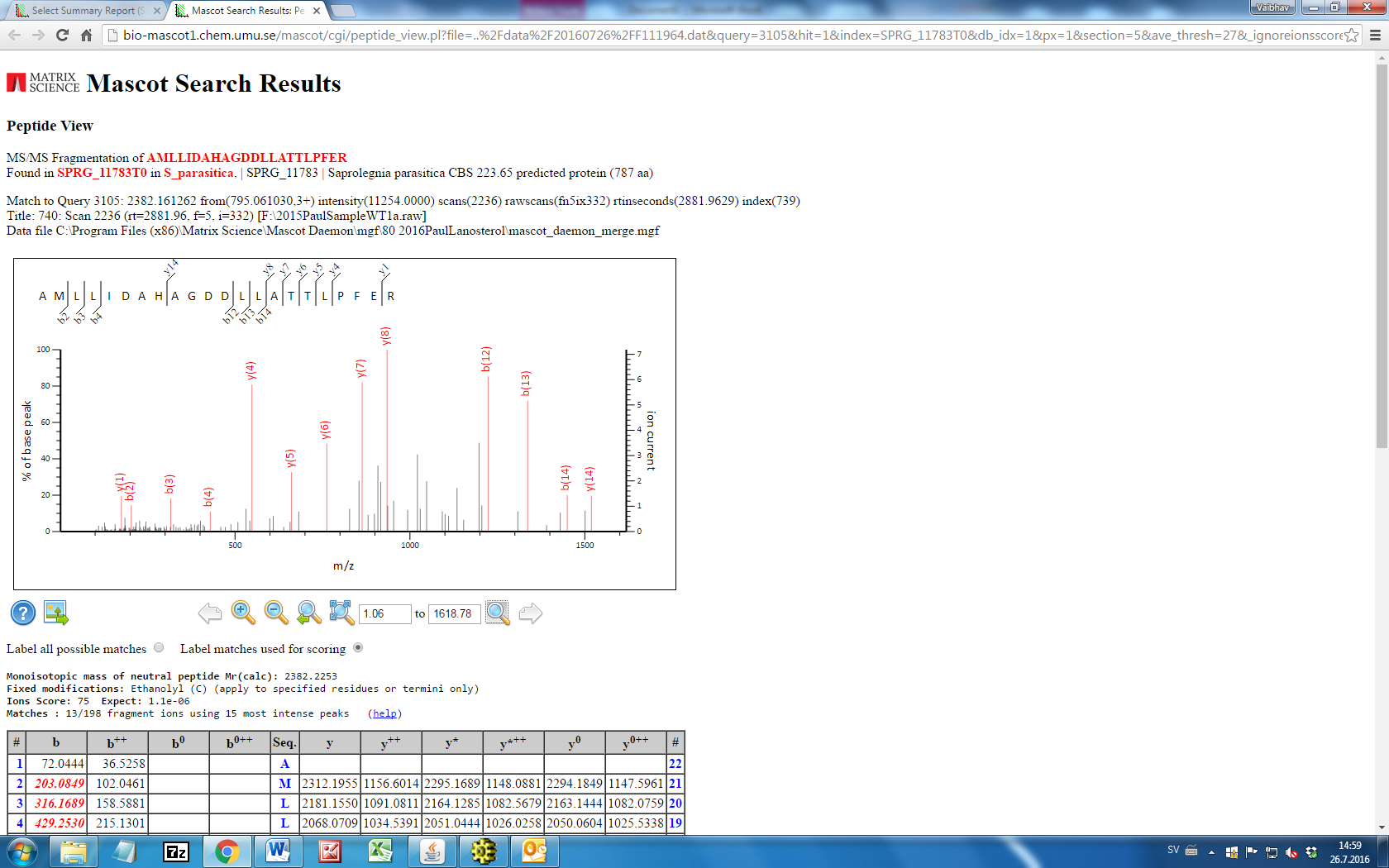


**Figure S3.** Assessment of the stability of 2,3-oxidosqualene (OS) in different media. The stability of OS was assessed in different media by incubating the substrate overnight at room temperature in (A) water, (B) LB medium and (C) *in vitro* reaction buffer. No peak corresponding to sterols or any other cyclised molecules became apparent after incubation in these different conditions.


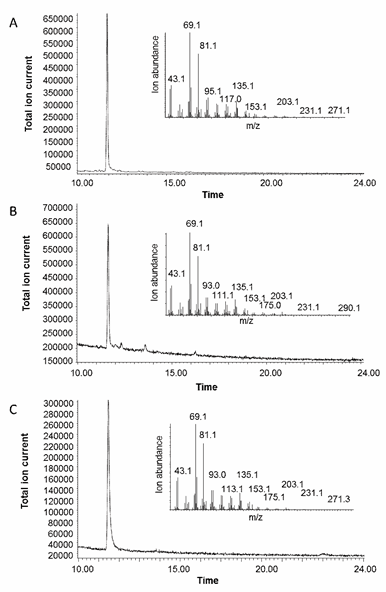


**Figure S4.** Amounts of 2,3-oxidosqualene (OS) and lanosterol (LA) recovered from *Sp*LASA enzyme assays by chloroform-methanol extraction. 10.7 µg OS was incubated in the presence of 37.5, 75.0 or 112.5 µg of *Sp*LASA protein in 1.5 mL reaction buffer. The total combined amount of LA and OS subsequently recovered by chloroform-methanol extraction was constant for all samples within a tolerable range of standard deviation. Cholesterol was added to samples prior to the extraction process as an internal standard for quantification calculations, to account for losses in the extraction process. All experiments were performed in triplicate.


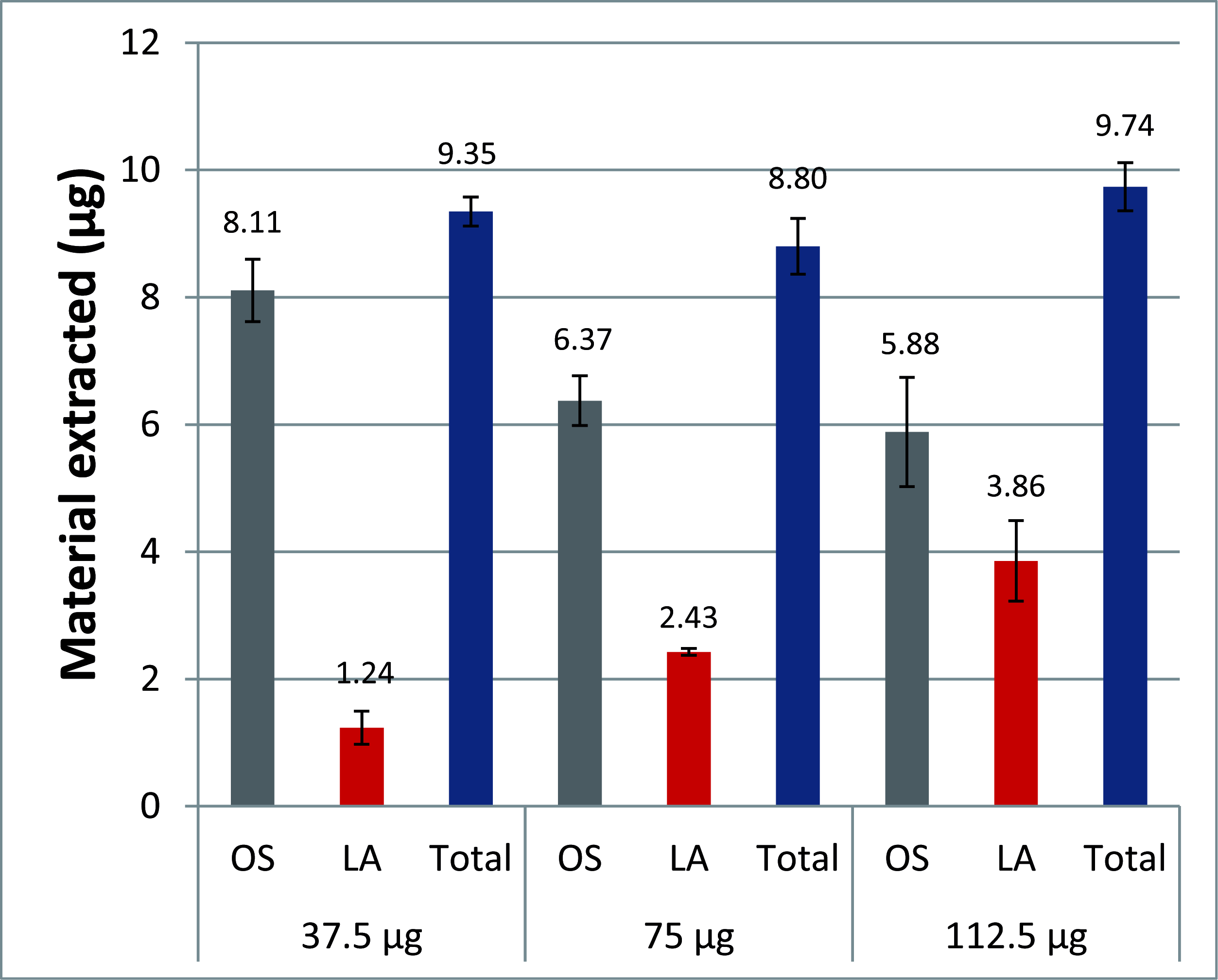

Supplement: Supplementary file 1 [file Data_Sheet_1.docx]
